# Thrombo-inflammation and Rethinking the Role of Aspirin in Kawasaki Disease

**DOI:** 10.1007/s11926-026-01211-5

**Published:** 2026-02-06

**Authors:** Begüm Kocatürk, Beyda Berberoğulları, Emil Aliyev, Erdal Sağ, Seza Özen, Moshe Arditi

**Affiliations:** 1 Department of Basic Oncology, Hacettepe University Cancer Institute, Ankara, Türkiye; 2 Department of Pediatrics, Division of Pediatric Rheumatology, Faculty of Medicine, Hacettepe University, Ankara, Türkiye; 3 Department of Pediatrics, Cedars-Sinai Guerin Children’s, Cedars-Sinai Health Sciences University, Los Angeles, CA USA; 4 Department of Biomedical Sciences, Infectious and Immunologic Diseases Research Center (IIDRC), Cedars-Sinai Health Sciences University, Los Angeles, CA USA; 5 Smidt Heart Research Institute, Cedars-Sinai Health Sciences University, Los Angeles, CA USA

**Keywords:** Kawasaki disease, Thrombosis, Coronary artery aneurysm, Aspirin, Anticoagulation

## Abstract

**Purpose of Review:**

Kawasaki Disease (KD) is a medium vessel vasculitis of young children that affects coronary arteries (CAs). The aim of therapy is to control inflammation and prevent coronary artery damage and alleviate thrombosis. This review aims to summarize the thrombosis-related complications and complementary therapy approaches to combat them. It also focuses on the ambiguity regarding the optimal aspirin dose in standard KD therapy.

**Recent Findings:**

KD patients with large or giant coronary artery aneurysms (CAAs) in particular face the risk of developing thrombosis. The standard therapy regimen during the acute phase consists of intravenous immunoglobulin (IVIG) plus aspirin. Although the dosing for IVIG is constant, the aspirin dose continues to be debated. The standard treatment with aspirin and IVIG might be adequate for individuals with small aneurysms whereas, patients suffering from large aneurysms and in a hypercoagulable state might need additional and extended therapy consisting of several antiplatelet reagents and anticoagulants. In case of thrombosis formation, thrombolytic therapy is also recommended.

**Summary:**

Cardiac and thrombotic problems are severe outcomes of KD. Patients need to be screened on a regular basis with echocardiography. The ideal aspirin dose during acute KD treatment is still controversial and while several recent studies pinpoint the superiority of low doses, there is no universal consensus. The variation in treatment response might be due to the different ethnic composition of study groups and inter-individual differences. Overall, close monitoring of patient response and finding the optimal treatment strategy is crucial.

## Introduction

KD is an acute inflammatory disease mainly affecting medium-sized arteries, in particular coronary arteries and the leading cause of acquired heart disease among children [1]. Although it was first described in 1974, the etiology of KD still remains elusive. The increased prevalence of the disease in Asian countries [2], the association of HLA-DRB1, -B5, -Bw51 and -Bw44 with KD susceptibility [3] and single nucleotide polymorphisms in FcγR2a, CASP3, HLA class II, BLK, ITPKC and CD40 genes [1] indicate the likelihood of a genetic predisposition. In addition, the incidence rate shows significant increment in specific areas and specific months which may point to the involvement of an infectious agent in disease etiology [4]. Indeed, the rise in absolute neutrophil and monocyte count and γδT cell activation pattern in patients’ peripheral blood clearly show the activation of innate and adaptive immune systems [5]. The young age of affected children suggests that individuals subsequently become immune to the infectious agent. Overall, the widely accepted postulation is that an unknown microorganism, likely a novel virus, affects infants with genetic susceptibility, thus triggering KD vasculitis [6]. This hypothesis is further supported by the fact that siblings have a higher risk of developing the disease and most sibling cases occur within a short period of each other [7].

The diagnosis of Kawasaki disease (KD) has been based on clinical signs, cardiac evaluation, and laboratory findings, as outlined in the American Heart Association (AHA) scientific statements published over the years [1, 8, 9]. Symptoms of complete KD are defined as the presence of persistent fever along with four or five of the following clinical features: bilateral nonexudative conjunctival injection, changes of the peripheral extremities, mucosal changes in the oropharynx, cervical lymphadenopathy and polymorphous maculopapular rash [1]. In addition to these common signs, the reactivation of the Bacillus Calmette-Guérin (BCG) vaccination scar is considered as an important clinical sign for KD [10]. However, because KD presents with variable clinical features, diagnosis can be challenging. Furthermore, its overlap with the manifestations of other febrile illnesses in children can further delay early recognition.

The goal of therapy is to reduce inflammation and prevent thrombosis, thus the common treatment modality for KD is the administration of IVIG and aspirin. However, 10%-20% of patients show resistance to IVIG treatment and remain febrile putting them at higher risk of developing coronary artery lesions (CALs) [11]. For IVIG-refractory patients, a second round of IVIG, intravenous methylprednisolone (IVMP), oral prednisone, infliximab or anakinra are recommended yet there is no consensus on their success rate or superiority of one over another [1].

### Long Term and Short Term Cardiac Complications

KD is the most common cause of acquired heart disease globally [12]. The course of the disease can be divided into three stages: systemic inflammation, coronary inflammation and coronary damage. Systemic inflammation is dampened over time whereas persistent coronary arteritis results in aneurysm formation. In some patients aneurysms show regression, whereas other individuals may face with coronary artery occlusion [13]. Larger aneurysms have a higher tendency to persist. These aneurysms are inclined to suffer from thrombotic complications and stenosis which in turn may cause myocardial ischemia. These patients’ major coronary artery segments and myocardial functions need to be examined by echocardiography on a regular basis to evaluate luminal dimensions, thrombosis or ventricular dysfunction. Other risk factors for KD-related myocardial ischemia are discussed in detail elsewhere [1].

Cardiovascular complications are the main cause of mortality in KD patients and their presence is a supportive criterion for KD diagnosis. The most common complications are coronary artery abnormalities such as coronary arteritis, arterial fistula, dilatation, coronary ectasia, stenosis, aneurysm and myocardial infarction (MI) [14]. Myocarditis is widely observed in the early phase of the disease; however, it subsides as patients receive anti-inflammatory treatment [1]. The pericardium and endocardium, including valves, may also be inflamed. During acute phase, hyperdynamic precordium, arrhythmia, tachycardia and diastolic dysfunction are detected [1, 15]. Moreover, thrombosis, endothelial cell necrosis and obliteration of vascular lumen is observed in the coronary arteries of patients dying 2–3 weeks after the onset of the disease [16]. Aortic root dilation is also detected in the early stages of the disease and shows association with aortic regurgitation [1]. Dilations resolve within 4 to 8 weeks in most patients, whereas large aneurysms may stay deformed. The regressed aneurysms do not always show full recovery. Thickened intima and impaired vasodilation capacity are observed in these vessels [17]. In extreme cases, concurrent loss of intima, media and elastica is detected. Although rare, during the acute phase quickly enlarging aneurysms may rupture subsequently leading to myocardial ischemia, pericardial tamponade or even death [1].

Long-term management begins with the end of the acute course and aims to alleviate thrombosis, MI and cardiovascular problems. Long-term complications may arise due to injured CAs, arteriosclerosis or atherosclerosis. Damaged endothelium due to acute vasculitis puts patients at risk of developing atherosclerosis [18]. Sudden death due to MI may happen many years later in KD patients complicated with CAA and stenoses. In fact, overlooked KD cases are held responsible for most of the MI cases in young adults. Non-occlusive thrombi in CAA with distal embolization and progression of calcified stenoses may also lead to non-ST-segment elevation myocardial infarction (NSTEMI)/unstable angina in the long term [1]. Notably, patients with regressed aneurysms may still suffer from distorted myocardial flow reserve and endothelial dysfunction in the distant future and thus still face the risk of myocardial ischemia [1]. The likelihood of developing these conditions increases if patients are not treated. Given this situation, it is clear that pediatricians should work closely with cardiologists for successful disease management.

### Changes in Platelet Numbers and Functions in Kawasaki Disease

Platelet count changes throughout the course of KD. Thrombocytosis is usually observed in the 2^nd^ to 3^rd^ week of the disease and platelet count drops by 4–6 weeks after onset [19]. Elevated platelet counts are also seen in the *Lactobacillus casei* cell wall extract (LCWE)-induced murine model of KD vasculitis [20]. In support of this notion, thrombopoietic factors increase both in patients and in the mouse model model of KD [19, 20]. Aside from increased platelet production, their clearance from circulation might be hampered due to the saturation of the reticuloendothelial system by immune complexes thus resulting in elevated platelet counts [21]. Moreover, platelet characteristics differ between KD patients and febrile controls. In particular, lower platelet distribution width and mean platelet volume in the febrile period of KD patients might be used as an extra supportive finding for differential diagnosis [22]. Higher peak platelet count was detected in patients with CALs [21] and IVIG resistance [23] implying its association with poor prognosis. Moreover, a higher platelet count following IVIG treatment shows positive correlation with the duration of coronary artery dilation [24]. Platelet number is related with the severity of heart vessel inflammation and abdominal aorta aneurysm in the mice model as well [20].

Thrombocytopenia might also be detected in the acute phase of the illness [25] hence, even if thrombocytosis serves as a supportive lab finding for KD diagnosis, low platelet count does not exclude the presence of the disease. Although the exact mechanism of thrombocytopenia is not known in KD patients, it is believed that disseminated intravascular coagulation [26], development of idiopathic thrombocytopenic purpura [27] or macrophage activation syndrome [28] might be the underlying cause. Intriguingly, KD patients with concomitant thrombocytopenia are also at higher risk of developing CALs and MI [26].

Alterations in platelet biology is also observed at the functional level. Platelet activation markers are upregulated in patients and mouse model of KD [29]. These changes are related to worse outcome; increased levels of β-thromboglobulin shows strong association with CAA formation [30] and platelet vascular endothelial growth factor (VEGF) levels of naive patients shows correlation with the severity of CAA [31].

### Thrombotic Complications of Kawasaki Disease

Thrombosis is considered to be a serious complication of KD and mostly occurs in the early phase, with a median time of 16 days after onset [32]. The left anterior descending artery is the branch where most of the thrombotic complication occurs thus needs to be examined vigilantly with echocardiography in long-term management [32]. In severe cases thrombotic complications and rupture may also be observed at other medium-sized arteries [33]. In particular, giant CAAs have a higher tendency to suffer from thrombosis. Indeed, a Z score > 10 and an absolute coronary artery diameter ≥ 8 is associated with higher thrombosis risk [1]. Therefore, these patients are recommended to use anticoagulants for an extended duration. Notably, males with giant CAAs are more prone to develop thrombosis and this might be attributed to lower shear stress and higher platelet activation profile [32]. In addition, genetic factors were found to be a risk factor for thrombosis as ITPKC rs7251246 T > C polymorphism is associated with thrombotic complications in KD children [34]. Thrombosis might lead to stenosis, MI, ischemia or recanalization [35]. Although recanalization might sustain blood flow, recanalized vessels are also susceptible to occlusion due to intimal thickening.

Platelets play a primary role in coagulation and as mentioned above, their count and activation profile differ in KD patients. Activated platelets are prone to form aggregates which in turn regulate coagulation. In line with this, platelet activation markers are further elevated in KD patients with thrombosis [36]. Circulating aggregates are observed in LCWE-induced KD mouse model [20] and KD patients and preserve their hyperaggregation state throughout acute and convalescent periods [21]. Thrombotic complications such as peripheral gangrene might be present in individuals [37] and thrombocytosis is associated with an increased risk of peripheral gangrene in KD patients [38]. A study by Dogan et al. suggested that the use of prostacyclin analogue might be beneficial for the treatment of KD complicated by peripheral gangrene [39].

On the other hand, thrombosis and inflammation are two interlinked processes. The inflammatory response in KD patients results in changes in vascular endothelial cells, platelets and the coagulation system which in turn triggers a hypercoagulable state. Indeed, D-dimer levels are not only upregulated [40] but also shows association with C-reactive protein (CRP) in KD [41]. Inflammatory cytokines trigger vascular endothelial cell damage and injured endothelium is a hallmark of KD. Intriguingly, damage markers remain elevated for 10–15 days following IVIG treatment [42]. Dysregulated endothelium results in the loss of hemostatic protection. Consequently, a pro-coagulant environment is formed where platelet-activating factors are released from endothelial cells. Indeed, von Willebrand factor (vWF) [43] and soluble thrombomodulin [44] levels are upregulated in KD. Infiltration of inflammatory cells to the vascular wall may also result in the loss of endothelial lining, which in turn leads to exposing collagen and decreases prostacyclin production in the injured endothelium. This damage can also cause a drop in platelet cyclic adenosine monophosphate levels, which may result in platelet aggregation. Dysfunctional endothelium is also responsible for impaired fibrinolytic response following KD [45].

The thrombotic state is supported by the distorted blood flow resulting from CAAs, stenosis and lower shear stress. Consequently, heart attack, stroke and even death may occur thus thrombotic occlusion of coronary arteries is a major risk factor for patients. In fact, thrombin-antithrombin III complex, a marker for intravascular coagulation, is upregulated in KD [44]. Upregulation in fibrinogen and D-dimer levels in KD compared to the healthy group is notable [46]. Moreover, patients with CAL and IVIG resistance have further elevation in D-dimer levels, implying the presence of a predisposition for thrombus formation [47, 48]. FVIII activity and fibrinogen levels are elevated, whereas the antithrombin III amount decreases, particularly during the acute phase [30].

Individuals developing a hypercoagulable state during the acute phase are predisposed to face IVIG resistance and several coagulation parameters might be used to assess the likelihood of IVIG non-responsiveness [49]. Paradoxically, prothrombin time (PT) and activated partial thromboplastin time (aPTT) are prolonged in KD and might be due to extensive consumption of coagulation factors or liver injury. Indeed, increased levels of serum liver enzymes, hypoalbuminemia, hyperbilirubinemia is more profound in IVIG resistant children [49]. In addition, IVIG administration shortens PT and aPTT remarkably [42].

### Prevention of Coronary Artery Thrombosis (thromboprophylaxis), Low-dose Versus High-dose Aspirin and Outcomes

Giant CAAs are major risk factors for thrombosis in KD patients. Therefore, judicious measures to diminish thrombosis risk are valuable. The routine treatment during the acute febrile period of KD is IVIG plus aspirin. However, it was not until 1984 that IVIG was considered as a potential treatment modality for KD [50]. Prior to this, aspirin alone was considered the standard treatment for KD as it exerts its effects through the inhibition of the cyclooxygenase (COX) enzyme, thus resulting in lower prostaglandin and thromboxane (TX) levels. In KD patients, TXA2 [51] and TXB2 [52] levels were found to be upregulated which served as one of the justifications for the use of aspirin. Indeed, the sole use of high-dose aspirin (HDA) (80–180 mg/kg/day) was shown to reduce CAA incidence [53].

The controversy over high-dose versus moderate- or low-dose aspirin (LDA) in the treatment of acute Kawasaki disease (KD) centers on balancing anti-inflammatory effects with safety and efficacy. Traditionally, HDA (80–100 mg/kg/day) has been used for its anti-inflammatory properties during the acute febrile phase, alongside intravenous immunoglobulin (IVIG) [54]. However, recent studies suggest that lower doses (30–50 mg/kg/day or even 3–5 mg/kg/day) may be equally effective in reducing inflammation and preventing coronary artery aneurysms [54–63], while minimizing side effects such as gastrointestinal irritation, liver enzyme elevations, and Reye syndrome risk [60, 64], (Table 1). Several retrospective and prospective studies, particularly from Japan and the U.S., have shown no significant difference in coronary artery outcomes between high and moderate-dose aspirin (MDA) groups [59, 60, 63, 65], (Table 1). As a result, practice patterns vary globally. It is also important to note that, while HDA is favored in some observational studies and clinical trials for clinical course such as fever duration, hospital stay, and IVIG resistance, there is no obvious benefit of HDA use for CAL formation. On the other hand, meta-analyses, which primarily focus on CAL as the endpoint, indicate that HDA does not offer an advantage over LDA in preventing CAL. Overall, there is still no universal consensus, highlighting the need for a large-scale, randomized controlled trial to definitively guide aspirin dosing in acute KD.

**Table 1:** Summary of studies comparing the the outcomes of higher and lower doses of aspirin treatment regimens in Kawasaki Disease. The types of studies are indicated with letters. a denotes observational studies, b denotes reviews with meta-analysis and c denotes clinical trials

Study	Treatment Regimen	Number of patients	Comparison (Acute Phase)	Outcome
Hayashi et al. [75] a	30–50 mg/kg/day aspirin vs No aspirin (3–5 mg/kg/day aspirin in case of CAL formation) **IVIG:** N/A	735	MDA + IVIG vs Only IVIG or LDA + IVIG	Higher dose aspirin use is not superior regarding the risk of CAL formation, IVIg response. No aspirin group is associated with shorter duration of fever
Chiang et al. [74] b	≥ 30 mg/kg/day aspirin vs 3–5 mg/kg/day aspirin or no aspirin **IVIG:** 2 g/kg	12182	HDA/MDA + IVIG vs LDA + IVIG or Only IVIG	Prescribing lower dose or no aspirin during the acute phase of KD is associated with a reduced incidence of CAL
Jia et al. [78] b	≥ 30 mg/kg/day aspirin vs 3–5 mg/kg/day aspirin or no aspirin **IVIG:** 2 g/kg as a single infusion or 400 mg/kg/day (4 days)	12176	HDA/MDA + IVIG vs LDA + IVIG or Only IVIG	Elevated doses of aspirin do not provide a significant reduction in CAL incidence. Receiving HDA is associated with shorter duration of fever while there is no significant difference regarding duration of hospital stay and IVIg resistance between two groups
Platt et al. [89] a	≥ 10 mg/kg/day aspirin vs < 10 mg/kg/day aspirin	260	HDA/MDA + IVIG vs LDA + IVIG	Lower dose of aspirin is not associated with an increased rate of CAL formation, recrudescent fever, and longer hospital stay thus is a suitable option for initial treatment
Wang et al. [90] a	40–50 mg/kg/day aspirin vs 30–39 mg/kg/day aspirin vs 20–29 mg/kg/day aspirin **IVIG:** No use / 1 g/kg / 2 g/kg	2369	3 aspirin dosage regimens are compared	Aspirin dose escalation does not improve IVIG response and CAL formation
Dallaire et al. [55] a	80 mg/kg/day aspirin vs 3–5 mg/kg/day aspirin **IVIG:** N/A	1213	HDA + IVIG vs LDA + IVIG	LDA is not associated with the increased risk of coronary artery abnormalities, IVIG resistance, and longer fever duration compared to HDA
Kim et al. [2] b	≥ 30 mg/kg/day aspirin vs 3–5 mg/kg/day aspirin **IVIG:** 2 g/kg	8456	HDA/MDA + IVIG vs LDA + IVIG	While the HDA/MDA group has a higher incidence of CAA, the LDA group has a higher IVIG resistance rate and longer duration of fever
Amarilyo et al. [54] a	80–100 mg/kg/day aspirin vs 3–5 mg/kg/day aspirin **IVIG:** 2 g/kg	358	HDA + IVIG vs LDA + IVIG	HDA group has no superiority over LDA group with regard to CAA and fever duration, however LDA group is associated with a shorter hospital stay
Saulsbury [62] a	80–100 mg/kg/day aspirin vs 3–5 mg/kg/day aspirin **IVIG:** 2 g/kg as a single infusion or 400 mg/kg/dose (4 days)	70	HDA + IVIG vs LDA + IVIG	HDA showed no significant benefit in shortening the duration of fever and CAA formation compared to LDA
Terai et al. [63] c	80–120 mg/kg/day aspirin vs 30–50 mg/kg/day aspirin **Total IVIG:** 100 mg/kg – 2 g/kg	1629	HDA vs HDA + IVIG vs MDA vs MDA + IVIG	Aspirin dose does not affect the prevalence of coronary artery abnormalities, whereas HDA leads to shorter duration of fever compared to LDA
Durongpisitkul et al. [57] a	> 80 mg/kg/day aspirin vs ≤ 80 mg/kg/day aspirin **IVIG:** > 1 g/kg	4151	HDA + IVIG vs LDA + IVIG	Higher doses of aspirin show no significant difference in CAA incidence compared to lower doses of aspirin in both short-term and long-term follow-up
Akagi et al. [65] c	100 mg/kg/day aspirin vs 30 mg/kg/day aspirin **IVIG:** None	60	HDA vs MDA	HDA is not recommended as an initial treatment option due to its tendency to cause liver toxicity, and inability to reduce the incidence of CAL, however HDA is associated with shorter fever duration
Suzuki et al. [91] a	> 50 mg/kg/day aspirin vs 30–50 mg/kg/day aspirin vs 5–30 mg/kg/day aspirin vs 3–5 mg/kg/day aspirin **IVIG:** N/A	82109	4 aspirin dosage regimens are compared	Lower dose of aspirin use shows association with higher proportion of CAA and longer hospital stay. 30–50 mg/kg/day aspirin administration increases IVIG resistance rate
Ito et al. [92] a	50 mg/kg/day aspirin vs 30 mg/kg/day aspirin **IVIG:** 2 g/kg	587	2 aspirin dosage regimens are compared	Higher doses of aspirin provide lower rate of IVIG resistance, although do not show significant difference in the incidence of coronary artery aneurysms
Zheng et al. [77] b	≥ 30 mg/kg/day aspirin vs 3–5 mg/kg/day aspirin **IVIG:** 2 g/kg	11103	HDA/MDA + IVIG vs LDA + IVIG	Lower dose of aspirin is not inferior to higher doses of aspirin regarding the incidence of CAL, and the duration of fever or hospital stay, and the incidence of IVIG resistance
Dhanrajani et al. [56] a	80–100 mg/kg/day aspirin vs 3–5 mg/kg/day aspirin **IVIG:** 2 g/kg	249	HDA + IVIG vs LDA + IVIG	LDA is linked with the increased rates of IVIG resistance compared to HDA. No significant difference is shown on the duration of hospital stay and the incidence of CAA
Yokoyama et al. [93] c	100–150 mg/kg/day aspirin vs 30 mg/kg/day aspirin vs	23	HDA vs. MDA	HDA administration is associated with a shorter duration of fever
Kuo et al. [79] a	80–100 mg/kg/day aspirin vs No aspirin **IVIG**: 2 g/kg	134	HDA + IVIG vs Only IVIG	HDA administration during the acute phase of KD can not reduce the incidence of CAL and IVIG resistance rate significantly
Safar et al. [61] a	≥ 30 mg/kg/day aspirin vs 3–5 mg/kg/day aspirin vs No aspirin **IVIG:** 2 g/kg or N/A	66126	HDA + IVIG vs LDA + IVIG or Only IVIG	There are no significant differences observed between 3 groups regarding CAA incidence, IVIG resistance rate, duration of fever and hospital stay
Huang et al. [59] a	80–100 mg/kg/day aspirin vs 30–50 mg/kg/day aspirin vs 3–5 mg/kg/day aspirin vs No aspirin **IVIG:** 2 g/kg	1944	Different doses of aspirin were compared	Higher dose of aspirin is not superior to lower doses of aspirin regarding CAL incidence, IVIG resistance rate, and duration of hospital stay
Sanati et al. [94] a	80–100 mg/kg/day aspirin vs No aspirin **IVIG**: 2 g/kg	62	HDA + IVIG vs Only IVIG	HDA administration does not confer any benefit regarding coronary artery abnormalities
Kwon et al. [60] a	80–100 mg/kg/day aspirin vs 30–50 mg/kg/day aspirin vs No aspirin **IVIG:** 2 g/kg	323	HDA + IVIG vs MDA + IVIG vs Only IVIG	Even though there is no significant difference in the incidence of coronary artery dilatations, duration of fever and hospitalization among groups, high-dose aspirin use is associated with Reye Syndrome while no aspirin use was associated with IVIG resistance
Huang et al. [58] a	30–50 mg/kg/day aspirin vs 3–5 mg/kg/day aspirin vs No aspirin **IVIG:** 2 g/kg	910	MDA + IVIG vs LDA + IVIG vs Only IVIG	There is no association between aspirin dosage and the development of CAL
Migally et al. [95] a	80–100 mg/kg/day aspirin For > 7 days vs For 1–7 days vs No aspirin **IVIG**: 2 g/kg or no IVIG	126	3 aspirin treatment plans were compared	The duration of HDA administration does not affect the CAA persistence
Kuo et al. [67] a	≥ 30 mg/kg/day aspirin vs No aspirin **IVIG:** 2 g/kg	851	MDA/HDA + IVIG vs Only IVIG	No significant differences were found between no aspirin group and aspirin group in CAL formation, IVIG resistance, and duration of hospitalization
Lee et al. [96] a	80–100 mg/kg/day aspirin vs No aspirin **IVIG:** 2 g/kg	180	HDA + IVIG vs Only IVIG	HDA has no impact on IVIG resistance and CAL formation, although it causes shorter duration of fever
Liu et al. [76] a	≥ 30 mg/kg/day aspirin vs < 10mk/kg/day **IVIG:** 2 g/kg	12258	HDA/ MDA + IVIG vs LDA + IVIG	LDA + IVIG is as effective as HDA/MDA + IVIG in preventing CAA. Higher doses of aspirin is associated with shorter duration of fever

There are also papers questioning the necessity of aspirin in KD treatment. Hsieh et al. and Kuo et al. demonstrated that the absence of aspirin in the treatment regimen during the acute phase did not affect IVIG resistance, incidence of CAAs and duration of fever [66, 67].

The common dosing for IVIG is 2g/kg and as mentioned above, thrombosis and inflammation processes are closely related; IVIG acts as an indirect anticoagulant by dampening inflammation and coagulation activation markers decrease following IVIG administration [42]. However, current treatment regimens vary in terms of the optimal dose for aspirin in KD treatment. At higher doses, it has anti-inflammatory effects, whereas at lower doses anti-platelet function is pronounced. In North America, the use of HDA (80–100 mg/kg per day) may be preferred during the acute phase [68] on the other hand, a moderate dose (30–50 mg/kg per day) is favored in Japan [69]. During the acute febrile phase, the AHA continues to recommend either moderate-dose (30–50 mg/kg/day) or high dose (80–100 mg/kg/day) aspirin, divided into multiple doses, alongside IVIG treatment [8]. Some clinicians choose to use higher doses of aspirin for 2 weeks regardless of patients’ fever status [70]. Once the patient is afebrile, the recommendation is to transition to LDA, typically 3-5mg/kg/day once a day, which is then continued for 6 to 8 weeks or longer depending on coronary artery involvement. If any abnormality is detected in the coronary arteries, then LDA is used until normal echocardiographic values are obtained. It is important to note that concomitant use of ibuprofen or other nonsteroidal anti-inflammatory drugs interfering with the cyclooxygenase pathway with aspirin should be avoided as it antagonizes the antiplatelet action of aspirin [71].

Previous studies had suggested the benefits of using high doses of aspirin in KD treatment (Table 1). However high doses may have potential side effects such as hepatic toxicity, anemia, sensorineural hearing loss, Reye’s syndrome and bleeding events may arise, especially during the subacute phase when albumin levels return to normal levels [67]. However, the superiority of HDA over MDA or vice versa is not clear; thus, recent studies and AHA also support the use of MDA in the acute phase [8]. There are additional possible concerns of HDA, as higher doses of aspirin inhibit cyclooxygenase in the vascular wall as well which may diminish prostacyclin levels, thus promoting platelet aggregation [72]. Decreased hemoglobin levels with HDA use is also observed and might result from elevated hepcidin levels leading to distorted iron metabolism [67]. Moreover, high doses of salicylate treatment, either alone or combined with IVIG augments TNF-α production, a critical cytokine in CAL formation, has been reported in the mouse model of KD [73]. The impeding effect of HDA with regard to anti-inflammatory action of IVIG was also recapitulated in human studies. Patients receiving HDA have a delayed decrease in C-reactive protein (CRP) levels following IVIG treatment [67].

These findings strongly suggest that administration of MDA, maybe more beneficial and thus better reflect the current practice. On the other hand, recent studies from Asian countries also suggest to start with LDA from the start [58, 59, 74–77] or even omit aspirin altogether [58, 59, 67, 74, 75, 78, 79], (Table 1). We await further studies to confirm that coronary complications are not increased with LDA. It should also be noted that the LDA studies are from Asia and have not been confirmed in Caucasian populations.

### Treatment of Coronary Artery Thrombosis with Antiplatelet Agents, Antithrombotic and Fibrinolytic Therapy in Kawasaki Disease

Early diagnosis and treatment of thrombi is crucial to reduce the risk of cardiac complications. Platelet-mediated thrombotic complications are widely seen during the course of KD and aspirin is the prime treatment of choice. The fact that thrombosis is still observed in individuals taking aspirin also underlines the presence of other platelet activation pathways. Therefore, it is clear that the addition of alternative antiplatelet reagents to standard therapy might be required. The presence of severe CALs requires the concomitant use of other antiplatelet reagents with aspirin [64]. In that sense, the adenosine diphosphate (ADP)-receptor antagonist clopidogrel and GP IIb/IIIa inhibitor abciximab show promising effects in KD treatment (Table 2). Patients who do not respond to standard therapy might also receive these medications as a secondary treatment. Indeed, the use of abciximab resulted in regression and even resolution in CAAs of non-responders [80]. In addition, the phosphodiesterase inhibitor dipyridamole was shown to increase peripheral blood flow in coronary arteries, which in turn may alleviate stasis and eventually thrombosis [81]. Individuals with aspirin intolerance or G6PD deficiency are in need of alternative therapies. In these cases, other antiplatelet reagents need to be administered rather than aspirin. In addition, liver injury is a rare complication of KD. A study by Gao et al. indicated that children receiving aspirin are predisposed to have exacerbated liver injury compared to patients administered clopidogrel, suggesting it as a safer treatment option in this subgroup [82].

**Table 2:** Summary of studies comparing the outcomes of anti-platelet treatment regimens in Kawasaki Disease

Study	Treatment Regimen	Number of patients	Comparison	Outcome
Liu et al. [97]	N/A	77	Clopidogrel + Aspirin vs Low-Molecular-Weight Heparin + Aspirin	Lower dose aspirin combined with clopidogrel is a safe and effective option in antithrombotic therapy for children with KD complicated by coronary artery aneurysms
Williams et al. [98]	**IVIG:** 2 g/kg as a single dose **Aspirin:** 80–100 mg/kg/day **Abciximab:** 0,25 mg/kg IV loading bolus dose followed by an infusion of 0,125 μg/kg/minute for 12 h	15	Abciximab + IVIG + Aspirin vs IVIG + Aspirin	The addition of abciximab to standard therapy led to a more significant reduction in coronary artery aneurysm diameter compared to standard therapy alone
McCandless et al. [99]	**IVIG:** 2 g/kg **Aspirin:** 80- 100 mg/kg/day **Abciximab:** 0,25 mg/kg IV loading bolus dose followed by an infusion of 0,125 μg/kg/minute for 12 h	18	Abciximab + IVIG + Aspirin vs IVIG + Aspirin	Large CAAs are successfully regressed with abciximab combined with standard therapy compared to standard therapy alone at 3–5 years of follow-up

It is believed that platelets are mainly responsible for the initiation of arterial thrombi. However, humoral clotting factors are also involved in thrombus formation in KD thus anticoagulants such as warfarin or low molecular weight heparin (LMWH) are recommended to be combined with the standard treatment regimen by the AHA. Indeed, combined use of anticoagulants with aspirin decreases MI incidence in patients with giant aneurysms [83]. Although warfarin is the prime choice if dosing and maintenance are problematic, LMWH can be used as an alternative. It provides a similar antithrombotic profile with more minor but fewer major bleeding problems. Of note, a transient decrease in antithrombin levels is widely observed in KD patients, which may hamper the success of LMWH treatment [30]. Under these circumstances, fresh-frozen plasma or antithrombin might be given to patients. Recent studies also suggest that rivaroxaban is a safe alternative and prevents thrombotic risk in KD patients with large CAAs [84].

The balance between coagulation and the fibrinolytic system is crucial for homeostasis. The hypercoagulable state in KD is reinforced by coagulation activation as well as fibrinolytic system malfunction. In fact, elevated levels of plasminogen activator inhibitor-1 (PAI-1) [85] and lower tissue plasminogen activator (tPA)/PAI-1 ratio in patients with CAL [86] and increment in euglobulin lysis time [30] highlight the distortion in fibrinolysis. A study by Horigome et al. suggested that KD patients with thrombotic complications benefit from intracoronary tPA administration [87]. The superiority of treating intracoronary thrombolysis with plasminogen activators is a matter of discussion: i) It resolves thrombus faster than intravenous coronary thrombolysis [88], ii) patients who did not benefit from heparin or warfarin respond well to this approach [87] iii) the prevalent abundance of fibrin meshwork in KD-associated thrombus may render fibrinolytic therapy desirable [3].

Overall, successful medical management of KD patients hinges on judicious use of anti-inflammatory, antithrombotic and fibrinolytic therapies. It is clear that this regimen requires attention for the extra risk of bleeding complications and the therapeutic armamentarium for KD with large/giant aneurysms involves the combination of several antiplatelet agents and anticoagulants. Furthermore, in serious cases, invasive revascularization procedures such as catheterization, CA bypass operations, might be preferred.

## Conclusion

KD is the main cause of acquired heart disease. Inflammation-driven thrombotic complications are extensively described for many diseases, including KD. Disruption of hemostasis leads to major drawbacks such as ischemia, stenosis, MI or even death highlighting the importance of therapies targeting the coagulation system. The use of aspirin is a part of standard therapy, whereas there is still no universal consensus on the recommended dosing regimen during acute KD. 2017 AHA guidelines suggest the use of HDA and MDA for treatment, while some studies show that LDA is not inferior to higher doses of aspirin. Importantly, for individuals with big/giant aneurysms is inadequate and treatment should be supplemented with other antiplatelet agents and anticoagulants. Thrombolytic therapy is another important part of the treatment and plasminogen activators are found at the center of this treatment modality. Overall, it is clear that prophylaxis is critical for KD patients accompanied with a hypercoagulable state.

## Key References

- Jone PN, Tremoulet A, Choueiter N, Dominguez SR, Harahsheh AS, Mitani Y et al. Update on Diagnosis and Management of Kawasaki Disease: A Scientific Statement From the American Heart Association. Circulation. 2024;150:e481-e500.
  - ○ This paper provides the current guidelines for the diagnosis and management of the Kawasaki disease.
- Noval RM, Kocaturk B, Franklin BS, Arditi M. Platelets in Kawasaki disease: mediators of vascular inflammation. Nat Rev Rheumatol. 2024.
  - ○ This article presents an in-depth summary of platelet alterations, platelet-mediated changes, and platelet-targeted therapies in Kawasaki disease.
- Peng Y, Yi Q. Incidence and timing of coronary thrombosis in Kawasaki disease patients with giant coronary artery aneurysm. Thromb Res. 2023;221:30-4.
  - ○ This study defines the timing and risk factors associated with thrombotic complications in Kawasaki disease.
- Kuo HC, Lo MH, Hsieh KS, Guo MM, Huang YH. High-Dose Aspirin is Associated with Anemia and Does Not Confer Benefit to Disease Outcomes in Kawasaki Disease. PLoS One. 2015;10:e0144603.
  - ○ This paper highlights the side effects of HDA use in the treatment of Kawasaki disease, particularly anemia and impaired inflammation suppression.
- Zheng X, Yue P, Liu L, Tang C, Ma F, Zhang Y et al. Efficacy between low and high dose aspirin for the initial treatment of Kawasaki disease: Current evidence based on a meta-analysis. PLoS One. 2019;14:e0217274.
  - ○ A comprehensive meta-analysis comprising 11103 patients demonstrating the lack of superiority of higher doses of aspirin with respect to CAL incidence, duration of fever, length of hospital stay and IVIG resistance.

## Data Availability

No datasets were generated or analysed during the current study.

## References

[1] McCrindle BW, Rowley AH, Newburger JW, Burns JC, Bolger AF, Gewitz M, et al. Diagnosis, treatment, and long-term management of Kawasaki disease: a scientific statement for health professionals from the American Heart Association. Circulation. 2017;135:e927–99. 28356445 10.1161/CIR.0000000000000484

[2] Kim GB, Yu JJ, Yoon KL, Jeong SI, Song YH, Han JW, et al. Medium- or higher-dose acetylsalicylic acid for acute kawasaki disease and patient outcomes. J Pediatr. 2017;184:125–9. 28043685 10.1016/j.jpeds.2016.12.019

[3] Agarwal SK, Agarwal S. Role of intracoronary fibrinolytic therapy in contemporary PCI practice. Cardiovasc Revasc Med. 2019;20:1165–71. 30685340 10.1016/j.carrev.2018.11.021

[4] Pinna GS, Kafetzis DA, Tselkas OI, Skevaki CL. Kawasaki disease: an overview. Curr Opin Infect Dis. 2008;21:263–70. 18448971 10.1097/QCO.0b013e3282fbf9cd

[5] Hara T, Yamamura K, Sakai Y. The up-to-date pathophysiology of Kawasaki disease. Clin Transl Immunol. 2021;10:e1284. 10.1002/cti2.1284 PMC8109476 33981434

[6] Nagata S. Causes of Kawasaki disease-from past to present. Front Pediatr. 2019;7:18. 30805322 10.3389/fped.2019.00018 PMC6371652

[7] Banday AZ, Bhattacharya D, Pandiarajan V, Singh S. Kawasaki disease in siblings in close temporal proximity to each other-what are the implications? Clin Rheumatol. 2021;40:849–55. 32776314 10.1007/s10067-020-05328-5 PMC7416658

[8] Jone PN, Tremoulet A, Choueiter N, Dominguez SR, Harahsheh AS, Mitani Y, et al. Update on diagnosis and management of Kawasaki disease: a scientific statement from the American Heart Association. Circulation. 2024;150:e481–500. 39534969 10.1161/CIR.0000000000001295

[9] Newburger JW, Takahashi M, Gerber MA, Gewitz MH, Tani LY, Burns JC, et al. Diagnosis, treatment, and long-term management of Kawasaki disease: a statement for health professionals from the Committee on Rheumatic Fever, Endocarditis and Kawasaki Disease, Council on Cardiovascular Disease in the Young. American Heart Association Circulation. 2004;110:2747–71. 15505111 10.1161/01.CIR.0000145143.19711.78

[10] Jindal AK, Pilania RK, Prithvi A, Guleria S, Singh S. Kawasaki disease: characteristics, diagnosis, and unusual presentations. Expert Rev Clin Immunol. 2019;15:1089–104. 31456443 10.1080/1744666X.2019.1659726

[11] Tremoulet AH, Best BM, Song S, Wang S, Corinaldesi E, Eichenfield JR, et al. Resistance to intravenous immunoglobulin in children with Kawasaki disease. J Pediatr. 2008;153:117–21. 18571548 10.1016/j.jpeds.2007.12.021 PMC2526555

[12] Pilania RK, Tremoulet AH, Prinja S, Dahdah N, Singh S. Kawasaki disease: the most common cause of acquired heart disease among children globally. Cardiol Young. 2025;1–3. 10.1017/S1047951125000459 39968873

[13] Kato H, Sugimura T, Akagi T, Sato N, Hashino K, Maeno Y, et al. Long-term consequences of Kawasaki disease. A 10- to 21-year follow-up study of 594 patients. Circulation. 1996;94:1379–85. 8822996 10.1161/01.cir.94.6.1379

[14] Kuo HC. Preventing coronary artery lesions in Kawasaki disease. Biomed J. 2017;40:141–6. 28651735 10.1016/j.bj.2017.04.002 PMC6136281

[15] Furukawa S, Matsubara T, Jujoh K, Yone K, Sugawara T, Sasai K, et al. Peripheral blood monocyte/macrophages and serum tumor necrosis factor in Kawasaki disease. Clin Immunol Immunopathol. 1988;48:247–51. 3390972 10.1016/0090-1229(88)90088-8

[16] Visi G, Spina F, Del DF, Manetti AC, Maiese A, La RRet al. Autoptic Findings in cases of sudden death due to Kawasaki Disease. Diagnostics (Basel). 2023;13. 10.3390/diagnostics13111831 PMC10252566 37296682

[17] Sugimura T, Kato H, Inoue O, Fukuda T, Sato N, Ishii M, et al. Intravascular ultrasound of coronary arteries in children. Assessment of the wall morphology and the lumen after Kawasaki disease. Circulation. 1994;89:258–65. 8281655 10.1161/01.cir.89.1.258

[18] Iemura M, Ishii M, Sugimura T, Akagi T, Kato H. Long term consequences of regressed coronary aneurysms after Kawasaki disease: vascular wall morphology and function. Heart. 2000;83(3):307–11. 10677411 10.1136/heart.83.3.307 PMC1729327

[19] Ishiguro A, Ishikita T, Shimbo T, Matsubara K, Baba K, Hayashi Y, et al. Elevation of serum thrombopoietin precedes thrombocytosis in Kawasaki disease. Thromb Haemost. 1998;79:1096–100. 9657430

[20] Kocaturk B, Lee Y, Nosaka N, Abe M, Martinon D, Lane ME, et al. Platelets exacerbate cardiovascular inflammation in a murine model of Kawasaki disease vasculitis. JCI Insight. 2023;8 37279077 10.1172/jci.insight.169855 PMC10443810

[21] Levin M, Holland PC, Nokes TJ, Novelli V, Mola M, Levinsky RJ, et al. Platelet immune complex interaction in pathogenesis of Kawasaki disease and childhood polyarteritis. Br Med J (Clin Res Ed). 1985;290:1456–60. 3922532 10.1136/bmj.290.6480.1456 PMC1415706

[22] Zheng X, Wu W, Zhang Y, Wu G. Changes in and significance of platelet function and parameters in Kawasaki disease. Sci Rep. 2019;9:17641. 31776411 10.1038/s41598-019-54113-1 PMC6881449

[23] Wei M, Huang M, Chen S, Huang G, Huang M, Qiu D, et al. A multicenter study of intravenous immunoglobulin non-response in Kawasaki disease. Pediatr Cardiol. 2015;36:1166–72. 25812827 10.1007/s00246-015-1138-0

[24] Zhang X, He Y, Shao Y, Hang B, Xu Z, Chu M. Factors affecting the duration of coronary artery lesions in patients with the Kawasaki disease: a retrospective cohort study. Pediatr Rheumatol Online J. 2021;19:96. 34174872 10.1186/s12969-021-00589-z PMC8236149

[25] Hara T, Mizuno Y, Akeda H, Fukushige J, Ueda K, Aoki T, et al. Thrombocytopenia: a complication of Kawasaki disease. Eur J Pediatr. 1988;147:51–3. 3338478 10.1007/BF00442611

[26] Niwa K, Aotsuka H, Hamada H, Uchishiba M, Terai M, Niimi H. Thrombocytopenia: a risk factor for acute myocardial infarction during the acute phase of Kawasaki disease. Coron Artery Dis. 1995;6:857–64. 8696530

[27] Singh S, Gupta D, Suri D, Kumar RM, Ahluwalia J, Das R, et al. Thrombocytopenia as a presenting feature of Kawasaki disease: a case series from North India. Rheumatol Int. 2009;30:245–8. 19444450 10.1007/s00296-009-0947-y

[28] Jin P, Luo Y, Liu X, Xu J, Liu C. Kawasaki disease complicated with macrophage activation syndrome: case reports and literature review. Front Pediatr. 2019;7:423. 31737585 10.3389/fped.2019.00423 PMC6838014

[29] Noval RM, Kocaturk B, Franklin BS, Arditi M. Platelets in Kawasaki disease: mediators of vascular inflammation. Nat Rev Rheumatol. 2024. 10.1038/s41584-024-01119-3 38886559

[30] Burns JC, Glode MP, Clarke SH, Wiggins J Jr., Hathaway WE. Coagulopathy and platelet activation in Kawasaki syndrome: identification of patients at high risk for development of coronary artery aneurysms. J Pediatr. 1984;105:206–11. 6235335 10.1016/s0022-3476(84)80114-6

[31] Ueno K, Nomura Y, Hashiguchi T, Masuda K, Morita Y, Hazeki D, et al. Platelet vascular endothelial growth factor is a useful predictor for prognosis in Kawasaki syndrome. Br J Haematol. 2010;148:285–92. 19793253 10.1111/j.1365-2141.2009.07922.x

[32] Peng Y, Yi Q. Incidence and timing of coronary thrombosis in Kawasaki disease patients with giant coronary artery aneurysm. Thromb Res. 2023;221:30–4. 36455387 10.1016/j.thromres.2022.11.014

[33] Hoshino S, Tsuda E, Yamada O. Characteristics and fate of systemic artery aneurysm after Kawasaki disease. J Pediatr. 2015;167:108–12. 25981909 10.1016/j.jpeds.2015.04.036

[34] Liu J, Yuan P, Pang Y, Su D. ITPKC polymorphism (rs7251246 T > C), coronary artery aneurysms, and thrombosis in patients with Kawasaki disease in a Southern Han Chinese population. Front Immunol. 2023;14:1184162. 37404818 10.3389/fimmu.2023.1184162 PMC10315485

[35] Orenstein JM, Shulman ST, Fox LM, Baker SC, Takahashi M, Bhatti TR, et al. Three linked vasculopathic processes characterize Kawasaki disease: a light and transmission electron microscopic study. PLoS ONE. 2012;7:e38998. 22723916 10.1371/journal.pone.0038998 PMC3377625

[36] Fu L, MacKeigan DT, Gong Q, Che D, Xu Y, Pi L, et al. Thymic stromal lymphopoietin induces platelet mitophagy and promotes thrombosis in Kawasaki disease. Br J Haematol. 2023;200:776–91. 36341698 10.1111/bjh.18531

[37] Tomita S, Chung K, Mas M, Gidding S, Shulman ST. Peripheral gangrene associated with Kawasaki disease. Clin Infect Dis. 1992;14:121–6. 1571415 10.1093/clinids/14.1.121

[38] Gomez-Moyano E, Vera CA, Camacho J, Sanz TA, Crespo-Erchiga V. Kawasaki disease complicated by cutaneous vasculitis and peripheral gangrene. J Am Acad Dermatol. 2011;64:e74–5. 21496687 10.1016/j.jaad.2010.04.029

[39] Dogan OF, Kara A, Devrim I, Tezer H, Besbas N, Ozen S, et al. Peripheral gangrene associated with Kawasaki disease and successful management using prostacycline analogue: a case report. Heart Surg Forum. 2007;10:E70–2. 17311768 10.1532/HSF98.20061151

[40] Imamura T, Yoshihara T, Yokoi K, Nakai N, Ishida H, Kasubuchi Y. Impact of increased D-dimer concentrations in Kawasaki disease. Eur J Pediatr. 2005;164:526–7. 15909182 10.1007/s00431-005-1699-7

[41] Zhang N, Yu L, Xiong Z, Hua Y, Duan H, Qiao L, et al. Kawasaki disease complicated by peripheral artery thrombosis: a case report and literature review. Pediatr Rheumatol Online J. 2022;20:77. 36064564 10.1186/s12969-022-00738-y PMC9444104

[42] Sakurai Y, Takatsuka H, Onaka M, Takada M, Nishino M. Persistent endothelial damage after intravenous immunoglobulin therapy in Kawasaki disease. Int Arch Allergy Immunol. 2014;165:111–8. 25401215 10.1159/000368402

[43] Irazuzta JE, Elbl F, Rees AR. Factor VIII related antigen (von Willebrand’s factor) in Kawasaki disease. Clin Pediatr (Phila). 1990;29:347–8. 2361346 10.1177/000992289002900613

[44] Mason WH, Takahashi M. Kawasaki syndrome. Clin Infect Dis. 1999;28:169–85. 10064222 10.1086/515133

[45] Albisetti M, Chan AK, McCrindle BW, Wong D, Vegh P, Adams M, et al. Fibrinolytic response to venous occlusion is decreased in patients after Kawasaki disease. Blood Coagul Fibrinolysis. 2003;14:181–6. 12632029 10.1097/00001721-200302000-00010

[46] Chen PS, Chi H, Huang FY, Peng CC, Chen MR, Chiu NC. Clinical manifestations of Kawasaki disease shock syndrome: a case-control study. J Microbiol Immunol Infect. 2015;48:43–50. 23927822 10.1016/j.jmii.2013.06.005

[47] Kong WX, Ma FY, Fu SL, Wang W, Xie CH, Zhang YY, et al. Biomarkers of intravenous immunoglobulin resistance and coronary artery lesions in Kawasaki disease. World J Pediatr. 2019;15:168–75. 30809758 10.1007/s12519-019-00234-6

[48] Masuzawa Y, Mori M, Hara T, Inaba A, Oba MS, Yokota S. Elevated D-dimer level is a risk factor for coronary artery lesions accompanying intravenous immunoglobulin-unresponsive Kawasaki disease. Ther Apher Dial. 2015;19:171–7. 25257673 10.1111/1744-9987.12235

[49] Shao S, Yang L, Liu X, Liu L, Wu M, Deng Y, et al. Predictive value of coagulation profiles for both initial and repeated immunoglobulin resistance in Kawasaki disease: a prospective cohort study. Pediatr Allergy Immunol. 2021;32:1349–59. 33694279 10.1111/pai.13495 PMC8451858

[50] Furusho K, Kamiya T, Nakano H, Kiyosawa N, Shinomiya K, Hayashidera T, et al. High-dose intravenous gammaglobulin for Kawasaki disease. Lancet. 1984;2:1055–8. 6209513 10.1016/s0140-6736(84)91504-6

[51] Hidaka T, Nakano M, Ueta T, Komatsu Y, Yamamoto M. Increased synthesis of thromboxane A2 by platelets from patients with Kawasaki disease. J Pediatr. 1983;102:94–6. 6401332 10.1016/s0022-3476(83)80300-x

[52] Fulton DR, Meissner C, Peterson MB. Effects of current therapy of Kawasaki disease on eicosanoid metabolism. Am J Cardiol. 1988;61:1323–7. 2454025 10.1016/0002-9149(88)91177-0

[53] Koren G, Rose V, Lavi S, Rowe R. Probable efficacy of high-dose salicylates in reducing coronary involvement in Kawasaki disease. JAMA. 1985;254:767–9. 4009911

[54] Amarilyo G, Koren Y, Brik SD, Bar-Meir M, Bahat H, Helou MH, et al. High-dose aspirin for Kawasaki disease: outdated myth or effective aid? Clin Exp Rheumatol. 2017;35(Suppl 103):209–12. 28079513

[55] Dallaire F, Fortier-Morissette Z, Blais S, Dhanrajani A, Basodan D, Renaud C, et al. Aspirin dose and prevention of coronary abnormalities in Kawasaki disease. Pediatrics. 2017;139 28562282 10.1542/peds.2017-0098

[56] Dhanrajani A, Chan M, Pau S, Ellsworth J, Petty R, Guzman J. Aspirin dose in Kawasaki disease: the ongoing battle. Arthritis Care Res. 2018;70:1536–40. 10.1002/acr.23504 29287309

[57] Durongpisitkul K, Gururaj VJ, Park JM, Martin CF. The prevention of coronary artery aneurysm in Kawasaki disease: a meta-analysis on the efficacy of aspirin and immunoglobulin treatment. Pediatrics. 1995;96:1057–61. 7491221

[58] Huang X, Huang P, Zhang L, Xie X, Xia S, Gong F, et al. Is aspirin necessary in the acute phase of Kawasaki disease? J Paediatr Child Health. 2018;54:661–4. 29271519 10.1111/jpc.13816

[59] Huang YH, Hsin YC, Wang LJ, Feng WL, Guo MM, Chang LS, et al. Treatment of Kawasaki disease: a network meta-analysis of four dosage regimens of aspirin combined with recommended intravenous immunoglobulin. Front Pharmacol. 2021;12:725126. 34456735 10.3389/fphar.2021.725126 PMC8397445

[60] Kwon JE, Roh DE, Kim YH. The impact of moderate-dose acetylsalicylic acid in the reduction of inflammatory cytokine and prevention of complication in acute phase of Kawasaki disease: the benefit of moderate-dose acetylsalicylic acid. Children (Basel). 2020;7 33081227 10.3390/children7100185 PMC7602855

[61] Safar FM, Kaabi WM, Aljudaibi RS, Alsaidi LM, Alharbi SS, Ibrahim AY, et al. The effectiveness of no or low-dose versus high-dose aspirin in treating acute Kawasaki Disease: a systematic review and meta-analysis. Clin Pract. 2024;14:1296–309. 39051299 10.3390/clinpract14040105 PMC11270423

[62] Saulsbury FT. Comparison of high-dose and low-dose aspirin plus intravenous immunoglobulin in the treatment of Kawasaki syndrome. Clin Pediatr Phila. 2002;41:597–601. 12403377 10.1177/000992280204100807

[63] Terai M, Shulman ST. Prevalence of coronary artery abnormalities in Kawasaki disease is highly dependent on gamma globulin dose but independent of salicylate dose. J Pediatr. 1997;131:888–93. 9427895 10.1016/s0022-3476(97)70038-6

[64] Newburger JW, Takahashi M, Burns JC. Kawasaki disease. J Am Coll Cardiol. 2016;67:1738–49. 27056781 10.1016/j.jacc.2015.12.073

[65] Akagi T, Kato H, Inoue O, Sato N. Salicylate treatment in Kawasaki disease: high dose or low dose? Eur J Pediatr. 1991;150:642–6. 1915517 10.1007/BF02072625

[66] Hsieh KS, Weng KP, Lin CC, Huang TC, Lee CL, Huang SM. Treatment of acute Kawasaki disease: aspirin’s role in the febrile stage revisited. Pediatrics. 2004;114:e689–93. 15545617 10.1542/peds.2004-1037

[67] Kuo HC, Lo MH, Hsieh KS, Guo MM, Huang YH. High-dose aspirin is associated with anemia and does not confer benefit to disease outcomes in Kawasaki disease. PLoS One. 2015;10:e0144603. 26658843 10.1371/journal.pone.0144603 PMC4686074

[68] Dajani AS, Taubert KA, Gerber MA, Shulman ST, Ferrieri P, Freed M, et al. Diagnosis and therapy of Kawasaki disease in children. Circulation. 1993;87:1776–80. 8491037 10.1161/01.cir.87.5.1776

[69] Kusakawa S, Tatara K. Efficacies and risks of aspirin in the treatment of the Kawasaki disease. Prog Clin Biol Res. 1987;250:401–13. 3423052

[70] Ho LGY, Curtis N. What dose of aspirin should be used in the initial treatment of Kawasaki disease? Arch Dis Child. 2017;102:1180–2. 29066520 10.1136/archdischild-2017-313538

[71] Catella-Lawson F, Reilly MP, Kapoor SC, Cucchiara AJ, DeMarco S, Tournier B, et al. Cyclooxygenase inhibitors and the antiplatelet effects of aspirin. N Engl J Med. 2001;345:1809–17. 11752357 10.1056/NEJMoa003199

[72] Hanley SP, Bevan J, Cockbill SR, Heptinstall S. Differential inhibition by low-dose aspirin of human venous prostacyclin synthesis and platelet thromboxane synthesis. Lancet. 1981;1:969–71. 6112387 10.1016/s0140-6736(81)91733-5

[73] Lau AC, Duong TT, Ito S, Yeung RS. Intravenous immunoglobulin and salicylate differentially modulate pathogenic processes leading to vascular damage in a model of Kawasaki disease. Arthritis Rheum. 2009;60:2131–41. 19565485 10.1002/art.24660

[74] Chiang MH, Liu HE, Wang JL. Low-dose or no aspirin administration in acute-phase Kawasaki disease: a meta-analysis and systematic review. Arch Dis Child. 2021;106:662–8. 33172886 10.1136/archdischild-2019-318245

[75] Hayashi K, Miyakoshi C, Hoshino S, Kobayashi N, Nakajima R, Sagawa H, et al. Initial intravenous immunoglobulin therapy without aspirin for acute Kawasaki disease: a retrospective cohort study with a Bayesian inference. BMJ Paediatr Open. 2024;8 38233084 10.1136/bmjpo-2023-002312 PMC10806463

[76] Liu K, Wang L, Gong L. High-dose or low-dose aspirin application in the initial phase of Kawasaki disease: a meta-analysis and systematic review. J Chem. 2022;2022:6303653.

[77] Zheng X, Yue P, Liu L, Tang C, Ma F, Zhang Y, et al. Efficacy between low and high dose aspirin for the initial treatment of Kawasaki disease: current evidence based on a meta-analysis. PLoS One. 2019;14:e0217274. 31117119 10.1371/journal.pone.0217274 PMC6531010

[78] Jia X, Du X, Bie S, Li X, Bao Y, Jiang M. What dose of aspirin should be used in the initial treatment of Kawasaki disease? A meta-analysis. Rheumatology (Oxford). 2020;59:1826–33. 32159800 10.1093/rheumatology/keaa050

[79] Kuo HC, Lin MC, Kao CC, Weng KP, Ding Y, Chen CJ, et al. Efficacy of Intravenous Immunoglobulin alone on coronary artery lesion reduction in Kawasaki Disease. medRxiv. 2024.

[80] Bachlava E, Loukopoulou S, Karanasios E, Chrousos G, Michos A. Management of coronary artery aneurysms using abciximab in children with Kawasaki disease. Int J Cardiol. 2016;220:65–9. 27372045 10.1016/j.ijcard.2016.06.062

[81] Kobayashi T, Sone K. Effect of dipyridamole on the blood flow in coronary aneurysms resulting from Kawasaki disease. Pediatr Cardiol. 1994;15:263–7. 7838798 10.1007/BF00798118

[82] Gao L, Wang W, Wang H, Xu Z, Zhou S, Geng Z, et al. The safety and effectiveness of clopidogrel versus aspirin in Kawasaki disease with mild-to-moderate liver injury. Sci Rep. 2023;13:18324. 37884573 10.1038/s41598-023-45647-6 PMC10603134

[83] Sugahara Y, Ishii M, Muta H, Iemura M, Matsuishi T, Kato H. Warfarin therapy for giant aneurysm prevents myocardial infarction in Kawasaki disease. Pediatr Cardiol. 2008;29:398–401. 18027010 10.1007/s00246-007-9132-9

[84] Montanez N, Sexson Tejtel SK, Menon NM. Pediatric thromboprophylaxis of large coronary artery aneurysm using rivaroxaban. J Pediatr Hematol Oncol. 2023;45:356–9. 37314881 10.1097/MPH.0000000000002690

[85] Senzaki H, Kobayashi T, Nagasaka H, Nakano H, Kyo S, Yokote Y, et al. Plasminogen activator inhibitor-1 in patients with Kawasaki disease: diagnostic value for the prediction of coronary artery lesion and implication for a new mode of therapy. Pediatr Res. 2003;53:983–8. 12621103 10.1203/01.PDR.0000061566.63383.F4

[86] Sakai M, Asayama K, Otabe T, Kohri T, Shirahata A. Low tissue plasminogen activator relative to plasminogen activator inhibitor-1 as a marker of cardiac complication in children with Kawasaki disease. Clin Appl Thromb Hemost. 2001;7:214–8. 11441982 10.1177/107602960100700306

[87] Horigome H, Sekijima T, Miyamoto T. Successful thrombolysis with intracoronary administration of tissue plasminogen activator in an infant with Kawasaki disease. Heart. 1997;78:517–8. 9415017 10.1136/hrt.78.5.517 PMC1892285

[88] Harada M, Akimoto K, Otaka M, Sato K, Oda H, Otsuki M, et al. Thrombolytic therapy in Kawasaki disease: a report of four cases. Pediatr Int. 2013;55:e111–5. 24134762 10.1111/ped.12130

[89] Platt B, Belarski E, Manaloor J, Ofner S, Carroll AE, John CC, et al. Comparison of risk of recrudescent fever in children with Kawasaki disease treated with intravenous immunoglobulin and low-dose vs high-dose aspirin. JAMA Netw Open. 2020;3:e1918565. 31899532 10.1001/jamanetworkopen.2019.18565 PMC6991313

[90] Wang J, Chen H, Shi H, Zhang X, Shao Y, Hang B, et al. Effect of different doses of aspirin on the prognosis of Kawasaki disease. Pediatr Rheumatol Online J. 2020;18:48. 32527316 10.1186/s12969-020-00432-x PMC7291457

[91] Suzuki T, Michihata N, Hashimoto Y, Yoshikawa T, Saito K, Matsui H, et al. Association between aspirin dose and outcomes in patients with acute Kawasaki disease: a nationwide retrospective cohort study in Japan. Eur J Pediatr. 2024;183:415–24. 37917176 10.1007/s00431-023-05302-8

[92] Ito Y, Matsui T, Abe K, Honda T, Yasukawa K, Takanashi JI, et al. Aspirin dose and treatment outcomes in Kawasaki disease: a historical control study in Japan. Front Pediatr. 2020;8:249. 32478021 10.3389/fped.2020.00249 PMC7241278

[93] Yokoyama T, Kato H, Ichinose E. Aspirin treatment and platelet function in Kawasaki disease. Kurume Med J. 1980;27:57–61. 7382423 10.2739/kurumemedj.27.57

[94] Sanati F, Bagheri M, Eslami S, Khalooei A. Evaluation of high-dose aspirin elimination in the treatment of Kawasaki disease in the incidence of coronary artery aneurysm. Ann Pediatr Cardiol. 2021;14:146–51. 34103852 10.4103/apc.APC_206_20 PMC8174624

[95] Migally K, Braunlin EA, Zhang L, Binstadt BA. Duration of high-dose aspirin therapy does not affect long-term coronary artery outcomes in Kawasaki disease. Pediatr Res. 2018;83:1136–45. 29554081 10.1038/pr.2018.44 PMC6019159

[96] Lee G, Lee SE, Hong YM, Sohn S. Is high-dose aspirin necessary in the acute phase of Kawasaki disease? Korean Circ J. 2013;43:182–6. 23613695 10.4070/kcj.2013.43.3.182 PMC3629244

[97] Liu YL, Wang XM, Chen TT, Shi K, Lu YH, Guo YH, et al. Clinical effect and safety of clopidogrel combined with aspirin in antithrombotic therapy for children with Kawasaki disease complicated by small/medium-sized coronary artery aneurysms. Zhongguo Dang Dai Er Ke Za Zhi. 2019;21:801–5. 31416506 10.7499/j.issn.1008-8830.2019.08.012 PMC7389908

[98] Williams RV, Wilke VM, Tani LY, Minich LL. Does abciximab enhance regression of coronary aneurysms resulting from Kawasaki disease? Pediatrics. 2002;109:E4. 11773572 10.1542/peds.109.1.e4

[99] McCandless RT, Minich LL, Tani LY, Williams RV. Does abciximab promote coronary artery remodeling in patients with Kawasaki disease? Am J Cardiol. 2010;105:1625–8. 20494673 10.1016/j.amjcard.2010.01.332

